# Large Outbreak of Guillain-Barré Syndrome, Peru, 2019

**DOI:** 10.3201/eid2611.200127

**Published:** 2020-11

**Authors:** César V. Munayco, Ronnie G. Gavilan, Gladys Ramirez, Manuel Loayza, Maria L. Miraval, Erin Whitehouse, Radhika Gharpure, Jesus Soares, Hans Vasquez Soplopuco, James Sejvar

**Affiliations:** Centro Nacional de Epidemiología Prevención y Control de Enfermedades, Lima, Peru (C.V. Munayco, G. Ramirez, M. Loayza);; Instituto Nacional de Salud, Lima (R.G. Gavilan, M.L. Miraval, H.V. Soplopuco);; Centers for Disease Control and Prevention, Atlanta, Georgia, USA (E. Whitehouse, R. Gharpure, J. Soares, J. Sejvar)

**Keywords:** Guillain-Barré syndrome, Campylobacter, Peru, outbreak, enteric infections, zoonoses, acute flaccid paralysis, bacteria

## Abstract

Outbreaks of Guillain-Barré syndrome (GBS) are uncommon. In May 2019, national surveillance in Peru detected an increase in GBS cases in excess of the expected incidence of 1.2 cases/100,000 population. Several clinical and epidemiologic findings call into question the suggested association between this GBS outbreak and *Campylobacter*.

Guillain-Barré syndrome (GBS) is the most common form of acute flaccid paralysis worldwide (*1*). It is characterized by motor weakness, areflexia, sensory abnormalities, and cytoalbuminologic dissociation in cerebrospinal fluid (*2*). An upper respiratory or gastrointestinal illness typically precedes GBS (*3*). *Campylobacter jejuni* infection is the most frequently identified precipitant of GBS and usually is associated with the acute motor axonal neuropathy form of GBS (*4*).

During the week of May 26, 2019, the Peruvian Ministry of Health surveillance system detected several cases of suspected GBS that exceeded the expected incidence of 1.2 cases/100,000 persons/year (i.e., 29 cases/year) (*1*). Since 2016, hospitals in Peru have reported suspected GBS cases to a passive surveillance system (https://www.dge.gob.pe/portal/index.php?option=com_content&view=article&id=653). In early May 2019, when the system was modified to an active surveillance system because of increasing incidence, the National Center of Epidemiology, Prevention, and Disease Control solicited cases. Examining physicians classified cases in accordance with the Brighton Collaboration case definition for GBS (*5*). The Instituto Nacional de Salud tested serum, urine, nasal swab samples, and feces for infectious pathogens using molecular panels for multipathogen detection (bioMérieux, https://www.biomerieux-diagnostics.com) and conventional microbiology assays.

During May 20–July 27, 2019, we identified 683 suspected or confirmed GBS cases in Peru. The largest outbreaks of GBS have involved »30–50 cases, except for large GBS outbreaks associated with Zika virus infection; thus, this outbreak was extremely unusual because of its size. Of the cases, 32 (6.9%) were Brighton level 1, 188 (27.5%) were Brighton level 2, and 463 (67.7%) were Brighton level 3. We classified Brighton levels 1 and 2 cases as confirmed, and Brighton level 3 cases as suspected (Figure; Appendix Figures 1, 2). Nine of Peru’s 24 departments reported GBS cases, which resulted in an annualized incidence of 30.9 cases/100,000 persons/year (Table).

**Figure:**
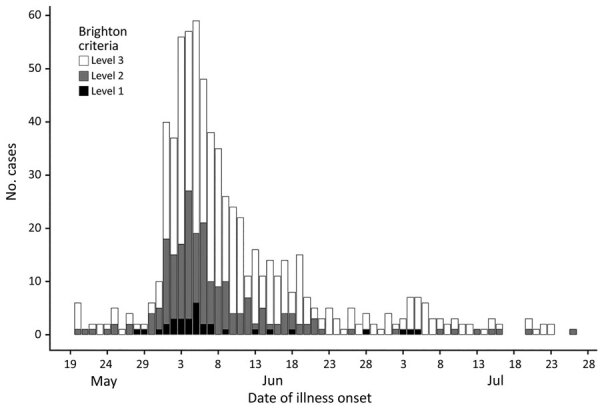
Cases of Guillain-Barré syndrome classified by Brighton Collaboration criteria (*5*) and date of illness onset, Peru, May–July 2019.

**Table:** General characteristics of persons with Guillain-Barré syndrome, Peru, 2019

Characteristic	No. cases	Population	Cases/100,000 population
Sex			
** M**	401	10,416,496	3.85
** F**	282	10,882,518	2.59
Age, y			
** 0–11**	48	4,321,980	1.11
** 12–17**	45	2,223,271	2.02
** 18–29**	118	4,498,823	2.62
** 30–59**	333	7,870,813	4.23
** >60**	139	2,384,127	5.83
Area			
** Junín**	132	1,389,850	9.50
** Piura**	115	1,901,896	6.05
** Cajamarca**	53	1,543,104	3.43
** La Libertad**	57	1,956,389	2.91
** Lambayeque**	31	1,300,720	2.38
** Lima**	247	10,458,367	2.36
** Callao**	25	1,067,815	2.34
** Huancavelica**	10	509,117	1.96
** Ancash**	13	1,171,756	1.11
** Total**	683	21,299,014	3.21
Peru, total	709	32,526,084	2.18

Of the 683 GBS patients, 287 (42.0%) had descending muscle weakness and 446 (65.3%) had ascending muscle weakness. Of 530 patients for whom data on antecedent illness were complete in the 4 weeks before neurologic symptom onset, 219 (41.3%) reported respiratory and gastrointestinal infections, 195 (36.8%) reported only a respiratory infection, 3 (0.6%) reported only a gastrointestinal infection, and 113 (21.3%) did not report any infection. Of 426 patients for whom hospitalization data were available, 64 (15.0%) required mechanical ventilation. Of 147 patients who had an electrodiagnostic exam, 100 (68.0%) had acute motor axonal neuropathy.

Clinical samples received by Instituto Nacional de Salud were as follows: serum (622 samples), urine (191), cerebrospinal fluid (230), nasal and pharyngeal swab samples (394), and feces (362) (Appendix Tables 1–3). We detected *Campylobacter* spp. in 19 (5.2%) fecal samples. Fecal cultures yielded 8 isolates confirmed as *C. jejuni* biotype I by Gram stain (*6*). Isolates were highly related by core-genome multilocus sequence typing and were sequence type 2993, Penner serotype HS:41.

This GBS outbreak was unusual because of the large number of cases. The incidence rate was nearly 25 times higher than expected (*1*) and higher than previously described GBS outbreaks. The rapid increase in numbers, followed by an equally precipitous decrease, might suggest a point-source exposure. The outbreak affected many geographically disparate regions, including some that differed substantially in geoclimatic properties.

General demographic features, such as slight male predominance and greater incidence with increased age, are typical for GBS (*7*). However, in many patients, a descending, rather than the more common ascending, paralysis developed (*8*). The clinical significance of this observation is unclear. Electrophysiologically, most cases appeared to have the acute motor axonal neuropathy phenotype of GBS, which has been closely associated with antecedent *C. jejuni* infection (*9*). 

PCR and culture detected the *C. jejuni* outbreak reported here. Genetic analysis confirmed the clonality of these isolates recovered from affected regions of Peru and identified genotype sequence type 2993, which has been associated with GBS outbreaks in China (*10*). These results support the hypothesis that this unprecedented GBS outbreak was related to an antecedent *Campylobacter* outbreak with point source. However, diarrheal illnesses shortly before or during the GBS outbreak were not reported; previous GBS outbreaks associated with *Campylobacter* mostly have occurred in the context of larger outbreaks of symptomatic diarrheal illness (*10*). Because of the wide distribution of outbreaks in many geographically separated regions, we questioned how all areas were exposed to *C. jejuni* within a short time frame. 

Limitations of our investigation included nonsystematic testing of samples and incomplete data on variables, such as hospitalization and clinical features. Epidemiologic investigations are ongoing to determine the potential antigenic source of the presumed infection, testing for *Campylobacter*-specific IgM and antiganglioside antibodies, additional isolate sequencing, and active surveillance for new cases.

Appendix Additional information about an outbreak of Guillain-Barré syndrome, Peru, 2019.
