# Supplementary material for: Large Outbreak of Guillain-Barré Syndrome, Peru, 2019
Source: Emerg Infect Dis. 2020 Nov;26(11):2778–80. doi: 10.3201/eid2611.200127 (PMC7588531; doi:10.3201/eid2611.200127)
Supplement: Appendix — Additional information about an outbreak of Guillain-Barré syndrome, Peru, 2019. [file 20-0127-Techapp-s1.pdf]

Article DOI: https://doi.org/10.3201/eid2611.200127

Large Outbreak of Guillain-Barré Syndrome, Peru, 2019

Appendix

Appendix Table 1. Laboratory tests and results of samples associated with Guillain-Barré syndrome, Peru, 2019*

Sample, laboratory test	Laboratory results		
	**No. tested**	**Positive**	**Negative**
Serum			
ELISA dengue IgM	240	2	238
qRT-PCR dengue	36	1	35
ELISA hepatitis E virus IgG	0	0	0
ELISA hepatitis E virus IgM	0	0	0
qRT-PCR Zika	135	0	135
ELISA Zika IgM	572	0	572
RT-PCR enterovirus	0	0	0
Urine: qRT-PCR Zika	53	2	51
Cerebrospinal fluid			
qRT-PCR Zika	46	0	46
ELISA IgM Zika	65	0	65
qRT-PCR dengue	1	0	1
ELISA IgM dengue	1	0	1
RT-PCR enterovirus	221	0	221
Enterovirus isolation	212	0	212
FilmArray meningitis and encephalitis	217	1	216
Nasal and pharyngeal swab samples			
IFD influenza A	165	0	165
qRT-PCR influenza A	202	1	201
IFD influenza B	165	1	164
qRT-PCR influenza B	202	1	201
qRT-PCR rhinovirus	179	11	168
qRT-PCR respiratory syncytial virus	179	5	174
IFD respiratory syncytial virus	162	0	162
qRT-PCR adenovirus	177	2	175
IFD adenovirus	162	0	162
IFD parainfluenza 1	162	0	162
IFD parainfluenza 2	162	0	162
IFD parainfluenza 3	162	0	162
IFD metapneumovirus	164	0	164
Fecal swab			
Campylobacter (FilmArray gastroenteritis)	361	19	342
qRT-PCR enterovirus	209	0	209
Enterovirus isolation	162	0	162
Other enteric bacteria (i.e., diarrheagenic *Escherichia coli*, *Shigella*, *Salmonella*)	362	44	318
Total	5,136	90	5,046

*IFD, isofraxidin; qRT-PCR, quantitative real-time reverse transcription.

Appendix Table 2. Number of other enteric bacteria isolated from fecal samples in an analysis of Guillain-Barré syndrome, Peru, 2019*

Other enteric pathogen	No.
Salmonella spp.	1
Enteroinvasive *Escherichia coli*	11
Enteropathogenic *E. coli*	1
Enterotoxigenic *E. coli*	32
Shigella spp.	2
Nondiarrheagenic *E. coli*	157
Total	204

Appendix Table 3. Number of samples received by Peruvian National Institute of Health in an investigation of Guillain-Barré syndrome, Peru, 2019

Samples	No. samples
Feces	275
Nasal and pharyngeal swabs	394
Fecal swab	322
Cerebrospinal fluid	230
Urine	191
Serum	622
Other	2
Total	2,036

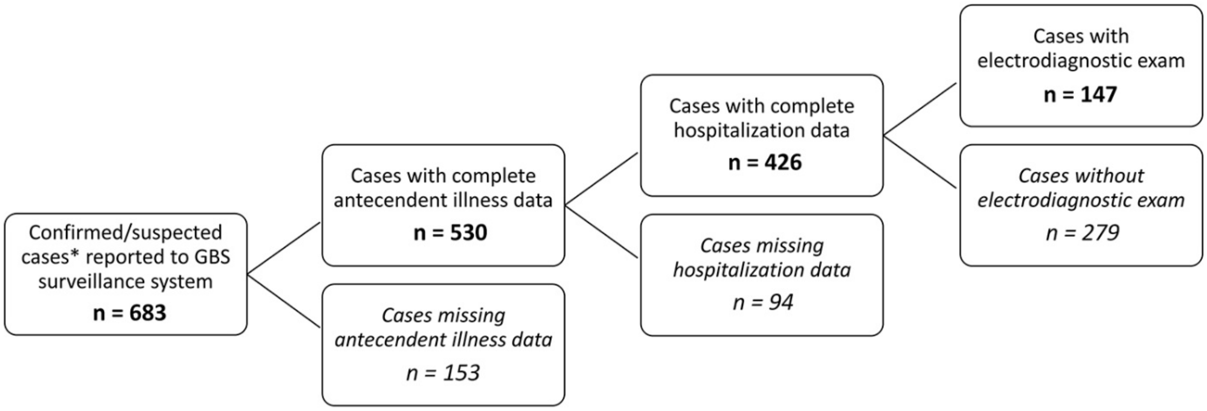

Appendix Figure 1. Flow diagram for enrollment of cases of GBS, Peru, 2019. *Brighton levels 1 and 2 cases (n = 220) were classified as confirmed. Brighton level 3 cases (n = 463) were classified as suspected. GBS, Guillain-Barré syndrome.

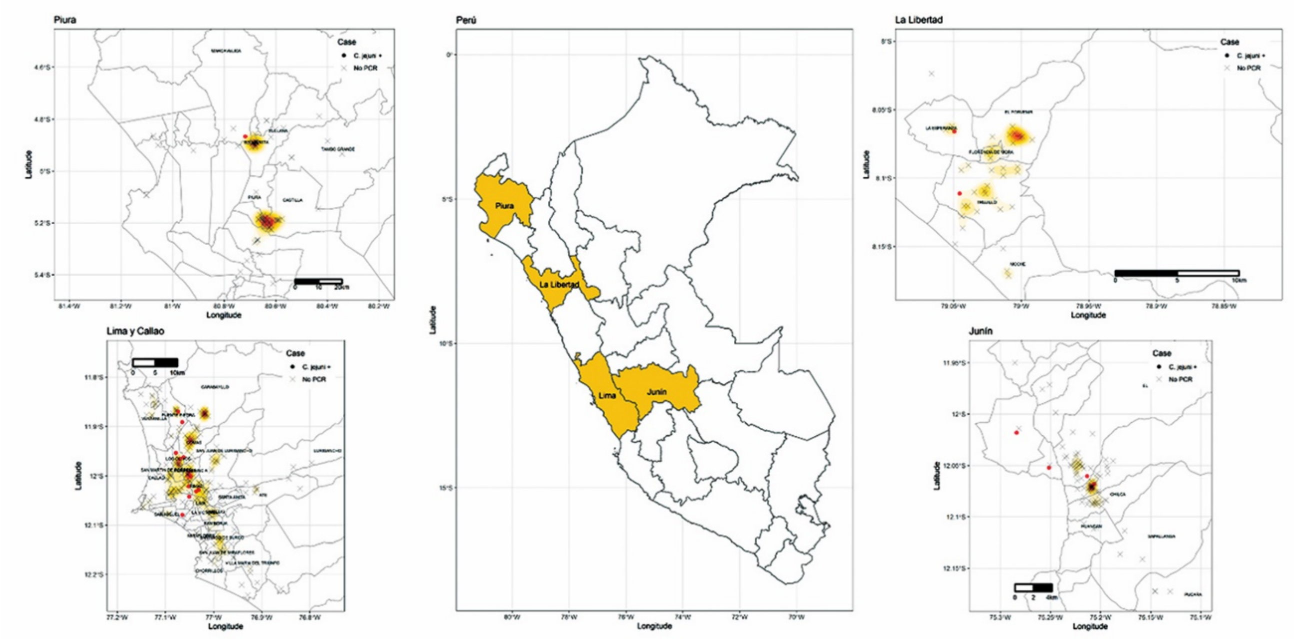

Appendix Figure 2. Spatial distribution of Guillain-Barré syndrome clusters detected by kernel density, Peru, 2019.
